# Sustainable Purchasing Practices: A Comparison of Single-use and Reusable Pulse Oximeters in the Emergency Department

**DOI:** 10.5811/westjem.58258

**Published:** 2023-09-25

**Authors:** Juliana Duffy, Jonathan E. Slutzman, Cassandra L. Thiel, Meghan Landes

**Affiliations:** * University of Toronto, Temerty Faculty of Medicine, Division of Emergency Medicine, Toronto, Ontario, Canada; † Massachusetts General Hospital, Center for the Environment and Health, Department of Emergency Medicine, Boston, Massachusetts; ‡ Harvard Medical School, Boston, Massachusetts; § NYU Langone Health, Department of Population Health, New York; ∥ University of Toronto, Temerty Faculty of Medicine, Division of Community and Family Medicine, Toronto, Ontario, Canada

## Abstract

**Background:**

Delivering healthcare requires significant resources and creates waste that pollutes the environment, contributes to the climate crisis, and harms human health. Prior studies have generally shown durable, reusable medical devices to be environmentally superior to disposables, but this has not been investigated for pulse oximetry probes.

**Objective:**

Our goal was to compare the daily carbon footprint of single-use and reusable pulse oximeters in the emergency department (ED).

**Methods:**

Using a Life Cycle Assessment (LCA), we analyzed greenhouse gas (GHG) emissions from pulse oximeter use in an urban, tertiary care ED, that sees approximately 150 patients per day. Low (387 uses), moderate (474 uses), and high use (561 uses), as well as cleaning scenarios, were modelled for the reusable oximeters and compared to the daily use of single-use oximeters (150 uses). We calculated GHG emissions, measured in kilograms of carbon dioxide equivalents (kgCO_2_e), across all life cycle stages using life-cycle assessment software and the ecoinvent database. We also carried out an uncertainty analysis using Monte Carlo methodology and calculated the break-even point for reusable oximeters.

**Results:**

Per day of use, reusable oximeters produced fewer greenhouse gases in low-, moderate-, and high-use scenarios compared to disposable oximeters: 3.9 kgCO_2_e, 4.9 kgCO_2_e, 5.7 kgCO_2_e vs 23.4 kgCO_2_e, respectively). An uncertainty analysis showed there was no overlap in emissions, and a sensitivity analysis found reusable oximeters only need to be used 2.3 times before they match the emissions created by a single disposable oximeter. Use phases associated with the greatest emissions varied between oximeters, with the cleaning phase of reusables responsible for the majority of its GHG emissions (99%) compared to the production phases of the single-use oximeter (74%).

**Conclusion:**

Reusable pulse oximeters generated fewer greenhouse gas emissions per day of use than their disposable counterparts. Given that the pulse oximeter is an ubiquitous piece of medical equipment used in emergency care globally, carbon emissions could be significantly reduced if EDs used reusable rather than single-use, disposable oximeters.

## INTRODUCTION

The effect of climate change on human health is vast and includes damaging social, economic, and psychological effects.^1^ These effects are related to increasing numbers of weather events worldwide. Those extreme weather events are a direct consequence of human-induced climate change.^1^ Emergency departments (ED) stand at the front line of the healthcare system, and there is substantial evidence linking climate events such as extreme heat, poor air quality, heavy rainfall, or climate-driven outcomes such as increasing exposure to vector-borne illness, food and housing insecurity, to surges in ED visits.^2^ Acute increases in the demand for emergency care also contributes to high ED volumes, prolonged boarding times, a strain on human resources, and adverse patient outcomes.^3^ Paradoxically, the delivery of healthcare requires expenditure of significant resources that result in the production of greenhouse gases (GHG), such as carbon dioxide in quantities that will inevitably contribute to further increases in global temperatures.^4^ By reducing their carbon dioxide emissions, EDs can decrease their contribution toward human-induced climate change and accrue both short- and long-term benefits for the communities they serve.

GHG emissions are reported in kilograms of carbon dioxide equivalents (kgCO_2_e), a measure that includes all gases with global warming potential. In Canada, emissions from the healthcare system have been estimated to be responsible for 33 million tons of kgCO_2_e, or 4.6% of the national total.^5^ GHG emissions in healthcare come directly from health facilities; these include anesthetic gases and boilers (referred to as Scope 1), purchased electricity (Scope 2), and indirectly generated GHG from the production and disposal of materials and equipment procured by the organization (Scope 3).^6^ Studies have shown that Scope 3 emissions account for four-fifths of the healthcare GHG footprint in the United States.^7^

Healthcare systems, including the ED, have the responsibility to deliver patient care efficiently, fairly, and safely. Not being attentive to the environmental impact associated with healthcare delivery and its consequence on human health violates these duties. Therefore, clinicians and hospital administrators must prioritize initiatives that will reduce emissions and lessen the negative effects of climate change on health. Fortunately, there is a growing body of evidence to support environmentally sustainable operational practices to achieve this end.^5^ ^,^ ^8^ ^–^ ^16^

One such method is life cycle assessment (LCA), a tool to quantify the environmental impact of a product or process from cradle to grave. LCA methodology has also been recommended to compare the carbon footprint of medical equipment,^17^ allowing healthcare organizations to make environmentally sustainable purchasing decisions. While LCAs have been carried out on numerous surgical devices,^8^ ^–^ ^10^ ^,^ ^13^ ^,^ ^16^ this methodology is uncommonly applied to materials in the ED setting.

Pulse oximeters, which are available in single-use or reusable forms, are a piece of medical equipment used in the ED (and other departments) to measure the oxygen saturation of a patient’s blood. They are small devices that are applied to the patient, most typically to a finger, for intermittent or continuous monitoring of vital signs and are used on every patient visiting the ED. As such a ubiquitous piece of medical equipment globally, oximeters represent a point of care opportunity to modify healthcare associated GHGs. To our knowledge, the carbon footprint of single-use and reusable oximeters has never been compared. We sought to determine the daily kgCO_2_e created in the production, transport, cleaning, and disposal of these two types of oximeters. This comparison will provide baseline data for EDs, and healthcare organization in general, seeking to make informed decisions on sustainable procurement practices for medical equipment.

## METHODS

### Setting

Our hospital is an urban, tertiary-care centre that provides ED services to approximately 55,000 patients annually, or 150 patients per day, in downtown Toronto, Ontario, Canada.^18^

### Study Scope

Life cycle assessment is a tool for mapping the environmental footprint of a product from its raw material extraction to eventual disposal. LCAs evaluate a product across several impact categories including potential to cause global warming, ozone depletion, smog, acidification (production of acid rain), eutrophication (over-accumulation of minerals and nutrients in a body of water), carcinogenics, respiratory effects, ecotoxicity, and fossil fuel depletion. This analysis focussed primarily on global warming potential measured in kgCO_2_e, although the remaining categories are included in the supplemental information.

An LCA begins with defining the functional unit, which allows products to be compared based on the service they provide. In this project, the functional unit was defined as one day of pulse oximetry measurement in the ED. Both reusable and disposable oximeters are approved for use in our hospital and are thus considered equal in terms of safety and functionality. Reusable oximeters are disinfected between use with a cleaning wipe that has been approved by the infection control service at our hospital and meets the instructions for use from the manufacturer.

**Figure 1.**
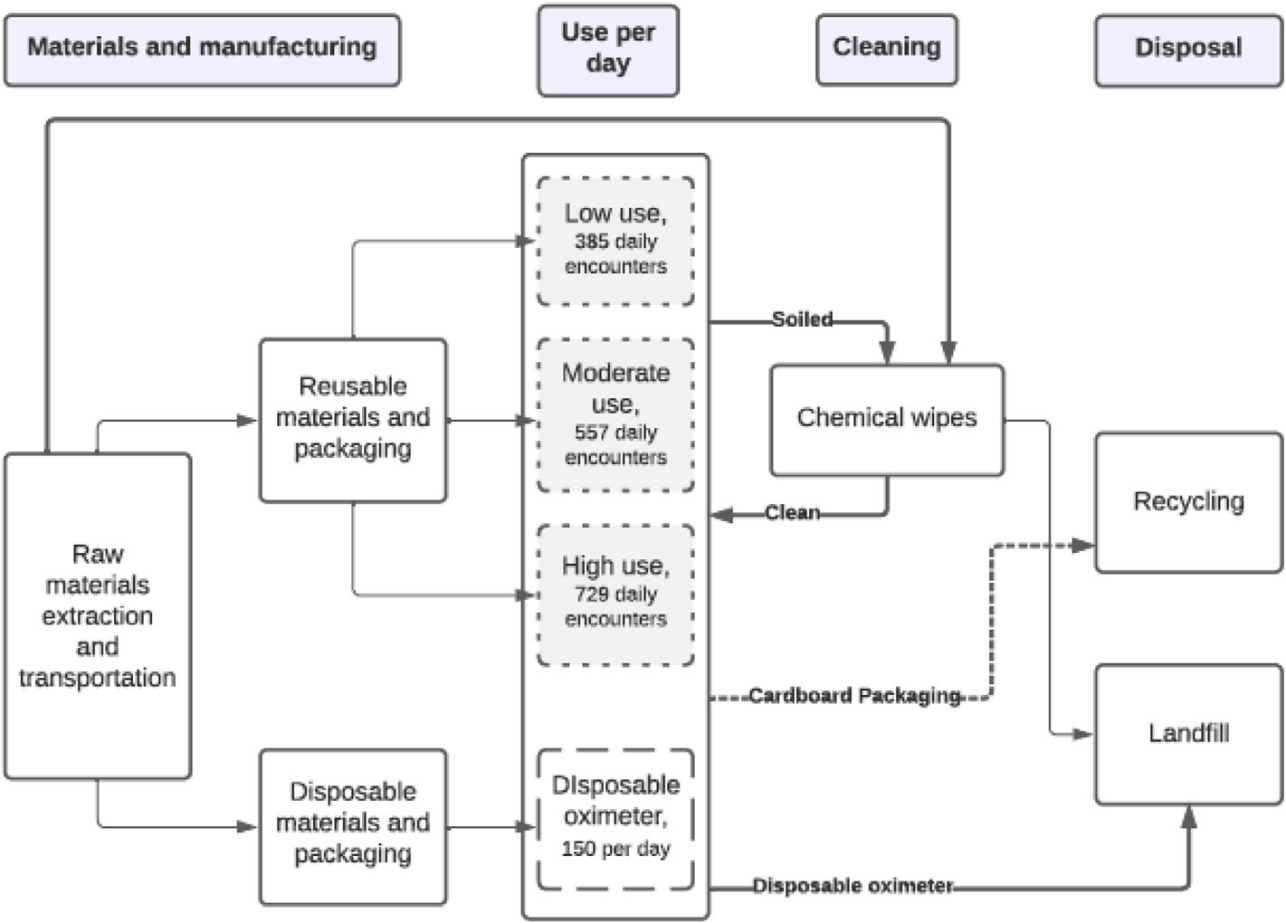
Process map for use of single-use vs disposable pulse oximeters.

Data for the reusable oximeter was scaled per use based on an estimated lifespan of one year, plus one cleaning wipe per patient encounter, and compared to the single-use disposable alternative. A more conservative lifespan was chosen for the reusable oximeter over the manufacturer’s estimate of two years^19^ to account for lost or damaged oximeters that would be replaced more frequently. The number of each type of oximeter making up the functional unit was determined by different use scenarios (see modelling parameters below). System boundaries included all materials used in the production of these devices and their associated cleaning products, as well as all energy required for their extraction, packaging, transport, and disposal (Figure 1).

Approval for this project was obtained from the University of Toronto Quality Improvement Review Committee (QI ID 20–0127).

### Materials and Manufacturing

We determined the composition and weight of each oximeter by obtaining materials information from the product manufacturers and then deconstructing each device to find the weight in grams of its individual parts. In instances where an exact description of the product’s materials was unavailable, such as in the case for LED sensors and cables in both reusable and disposable oximeters, we extrapolated from a similar product by a different manufacturer.^20^ Since we were unable to obtain the weights of the individual materials comprising these two items (LED sensor and cable), we assumed the total weight was divided evenly across all their materials. Notably, we excluded the gold plating that covered the pin header of the cable for the following reasons: its contribution was marginal relative to the other materials; it is present in equal amounts on both devices; we could not reliably estimate its weight; and its high global warming impact ran the risk of significantly altering our findings if the estimated weight were to be improperly calculated. Additionally, the power source to operate each oximeter was drawn in via their cables and was assumed to be equal between devices; therefore, it was also omitted from analysis.

We also included the cleaning wipes used to disinfect the reusable oximeters between each use. This information was collected from the manufacturer’s label as well as the material safety data sheet. The generic material compositions of each oximeter and the cleaning wipes are shown in Table 1. As one wipe is often used to disinfect multiple pieces of equipment simultaneously, we modeled one quarter of a wipe and its active ingredients per use. This decision, which was based on direct observation of our staff disinfecting equipment between uses, is in keeping with methodology described in a similar LCA.^8^ Since disinfection of pulse oximeters is the same for patients on advanced isolation precaution, we did not need to account for additional cleaning materials used for those patients.

**Table 1.** Material composition of single-use pulse oximeters, reusable oximeters, and cleaning wipes.

Single-use oximeter	Mass (g)
Packaging	24
Shield	0.08
Sensor top/bottom	1.13
LED sensor	0.07
Cable	15.99
TOTAL	41.27
**Reusable oximeter**	**Mass (g)**
Packaging	26.4
Spring	1.43
Plastic housing	10.75
Detector frame and pad	4.49
LED sensor	0.07
Cable	26.4
TOTAL	69.54
**Cleaning wipe**	**Mass (g)**
Ammonium chloride	0.0053
Isopropyl alcohol	0.575
Cotton fiber	0.25
Packaging	2.55
TOTAL	3.38

*g*, gram.

### Packaging and Transport

According to the manufacturer, single-use oximeters were packaged as 24 sensors per box and 20 boxes per large carton, whereas the reusable oximeters were packaged one per box and 20 boxes per large carton.^19^ We also included individual wrapping around each device. Both devices were manufactured in Tijuana, Mexico, and shipped by truck to Toronto,^19^ a distance of approximately 4,180 kilometers (km). As per the manufacturers of the cleaning wipes, there were 160 wipes per container and four containers per box.^19^ Cleaning wipes were manufactured in Michigan and shipped by truck to Toronto,^19^ approximately 408 km. Packaging and transport were also included and calculated per one-fourth of a wipe.

### Modelling Parameters

Every patient who registered in the ED had their pulse oximetry measured at least once, at triage. Then, depending on their acuity or the clinical scenario, patients may have no further oximetry readings, intermittent or continuous monitoring. Typically, disposable pulse oximeters were used by a single patient throughout the duration of their ED visit and were discarded at discharge. In our setting, this equaled 150 disposable oximeters per day. Alternatively, reusable oximeters were used on multiple patients over the course of a day and cleaned between each encounter. Our department has 34 reusable oximeters divided among the following locations: one in triage; 21 stationary machines located in patient rooms; and 12 attached to portable vital signs machines. Therefore, we compared the GHG emissions associated with 150 disposable oximeters to 34 reusable oximeters. Although the manufacturer’s estimated lifespan of the reusables was two years, we conservatively estimated it to be 365 days to account for lost or damaged oximeters that would be replaced more often. As a result, we compared 1/365^th^ of the manufacturing, transportation, and disposal impacts of our 34 reusable oximeters to 150 single-use alternatives.

To account for the variable number of disinfectant wipes consumed per day, we added the total daily usage for each of the 34 reusable oximeters (Table 2 and Figure 1). For the triage and stationary oximeters, there were a fixed number of daily uses. The single oximeter at triage was used and cleaned 150 times per day, once for every patient visiting the ED (150 uses). The 21 stationary oximeters were used an average of three times each per day (63 uses). This was based on the assumption that each of the 21 monitored rooms was filled with a new patient every eight hours, as the average ED length of stay in Ontario is 7.8 hours.^21^ Finally, for the remaining 87 patients not triaged to a monitored room, the frequency of pulse oximetry readings was highly variable. Therefore, we modeled three use scenarios (low-, moderate-, and high-frequency use) in which different proportions of those 87 patients had their vital signs checked every two hours over an eight-hour visit (Table 2). In the low-use scenario, we assumed that 29 patients (33%) had their vital signs repeated four times, whereas the remaining 58 patients (66%) of patients had their vital signs repeated only once. In the moderate-use scenario, we assumed that 58 patients (66%) had their vital signs repeated four times and 29 (33%) only once. Finally, in the high-use scenario we assumed that all 87 patients had their vital signs repeated four times.

**Table 2.** Daily use of reusable oximeters by emergency department location.

Uses per day	Location			Total
	Triage oximeter (n = 1)	Stationary oximeters (n = 21)	Portable oximeters (n = 12)	
Low use	150	63	174	387
Moderate use	150	63	261	474
High use	150	63	348	561

### Waste Management

Based on observations of disposal practices, all oximeters, sanitizing wipes, and plastic packaging were modeled as going into municipal waste, whereas cardboard packaging was recycled.

### Life Cycle Assessment Modeling

We performed LCA modeling using SimaPro v9.2.0.2 (PRé Consultants, Amersfoort, The Netherlands). We created a life cycle inventory (LCI) in SimaPro by matching materials and processes to those available in the ecoinvent 3.8 database (ecoinvent Association, Zurich, Switzerland).^22^ Detailed LCI data and unit process metadata are provided in the supplemental information (Tables 1–4). Environmental impact assessments were carried out using the US Environmental Protection Agency Tool for the Reduction and Assessment of Chemicals and other environmental Impacts (TRACI) 2.1 V1.06/US-Canada 2008 method.^23^ All software, databases, and models employed in this study are widely described and accepted by international standards and guidance.^24^ ^–^ ^26^

Once the LCA was prepared, we calculated the GHG emissions per day of both devices. For the single-use oximeters, this was simply the GHG emissions from the life cycle phases of production, packaging/transport, and disposal, multiplied by 150 uses per day. For the reusable oximeter, we multiplied its GHG emissions by 34 (to account for all oximeters in our ED) and divided this number by 365 to determine emissions per day. We then added the emissions created by the cleaning wipes to model total emissions by low-, moderate-, and high-use cleaning scenarios. Therefore, the emissions created by the production, packaging/transport, and disposal of the cleaning wipes are represented as the “cleaning phase” for the reusable oximeter.

We performed an uncertainty analysis using Monte Carlo methodology to account for the uncertainty inherent in LCI data and to appreciate the range of potential environmental impacts associated with each type of oximeter. The process of this analysis has been well described in previous literature.^10^ In this project, we calculated the resulting distribution from 1,000 random samplings. A 95% confidence interval as well as the median and standard deviation for GHG emissions created by disposable and reusable oximeters (including low-, moderate-, and high-use cleaning) was calculated. We then compared oximeters by life cycle phase (production, transport, cleaning, and disposal) to determine which phases created the highest emissions. Finally, a sensitivity analysis was carried out to estimate the number of reuses needed to make emissions from a reusable oximeter equivalent to the single-use oximeter. This is also known as a break-even analysis.

## RESULTS

### Life Cycle Assessment

**Figure 2.**
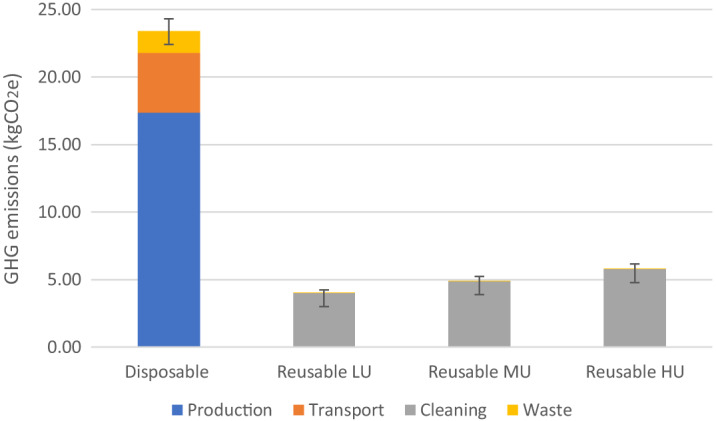
Greenhouse gas emissions per day of use by oximeter type. *kgCO*_2_, kilograms of carbon dioxide; *LU*, low-use cleaning scenario (n = 387); *MU*, moderate-use cleaning scenario (n = 474); *HU*, high-use cleaning scenario (n = 561). Error bars represent a 95% confidence interval from Monte Carlo analysis.

The global warming impact for the production, transport, cleaning, and disposal of reusable and disposable pulse oximeters is displayed in Table 3 and Figure 2. When these devices were compared per day of use (150 disposable oximeters, 34 reusable oximeters), reusable oximeters produced fewer greenhouse gases per day in low-, medium- and high-use scenarios compared to disposable oximeters (3.9 kgCO_2_e, 4.9 kgCO_2_e, 5.7 kgCO_2_e vs. 23.4 kgCO_2_e, respectively). This pattern was consistent across every major impact category (See supplemental Tables 1–3), and the results of the uncertainty analysis showed there was no overlap in emissions (Figure 2). To further contextualize this difference, over the duration of one-year, single-use oximeters create between 6,461–7,117 more kgCO_2_ than reusable oximeters within our department.

**Table 3.** Global warming impact or greenhouse gas emissions of pulse oximeters per day of use.

	Global warming impact (kgCO_2_e)			
Oximeter	Single use	Reusable		
		LU	MU	HU
Production	17.35	0.026	0.026	0.026
Transport	4.44	0.0046	0.0046	0.0046
Waste disposal	1.62	0.00219	0.00219	0.00219
**Cleaning wipes**				
Production	-	2.28	2.79	3.30
Transport	-	0.091	0.112	0.133
Waste disposal	-	1.59	1.95	2.30
**Total**	23.41	3.96	4.85	5.73

*kgCO*_2_, kilograms of carbon dioxide; *LU*, low-use cleaning scenario, reusable (n = 385); *MU*, moderate-use cleaning scenario, reusable (n = 557); *HU*, high-use cleaning scenario, reusable (n = 729).

For each device, there were vast differences between which phase of the life cycle contributed the most to GHG emissions per day (Figure 2). The cleaning phase of the reusable oximeter produced most of its GHG emissions (99%) followed by production (0.79%), transport (0.14%), and disposal (0.07%). Alternatively, the production phases of the single-use oximeter had the highest contribution (74%), followed by transport (18.9%) and disposal (6.9%). There was no cleaning phase for the single-use device.

Outside the cleaning scenario, the electric cables on the disposable and reusable oximeters had the highest carbon footprint, 0.073 and 0.144 kgCO_2_, respectively. (See Supplemental Tables 1 and 2).

**Figure 3.**
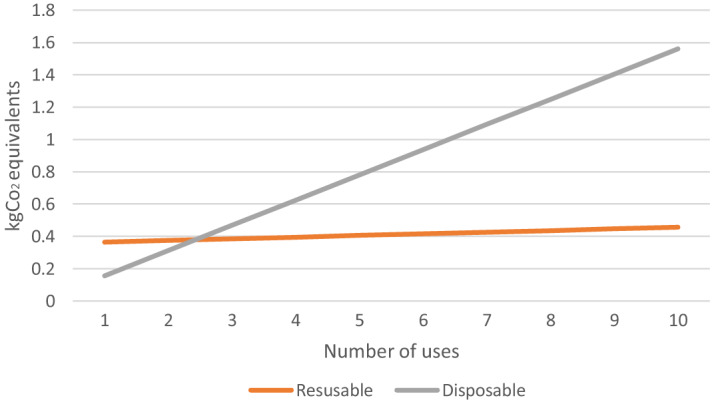
Break-even point of single-use and reusable oximeters. *kgCO*_2_, kilograms of carbon dioxide.

The results of our sensitivity analysis showed that reusable oximeters produce fewer emissions than disposable oximeters after only 2.3 uses per day with cleaning (Figure 3).

## DISCUSSION

In this study, we sought to calculate the environmental impact of single-use vs reusable oximeters in an urban ED. We found that reusable pulse oximeters, regardless of how frequently they were used and cleaned in the ED, created a significantly lower quantity of GHGs per day than is the case for single-use oximeters. This finding was consistent across all major environmental impact categories including ozone depletion, smog, acidification, eutrophication, carcinogenics, non-carcinogenics, respiratory effects, ecotoxicity, and fossil fuel depletion. (See Supplemental Information). Depending on the frequency that reusable oximeters were cleaned (high vs low frequency), the daily emissions created by disposable oximeters was three- to five-fold higher than those created by reusable oximeters in high- vs low-frequency use scenarios. Alternatively, reusable oximeters only needed to be used 2.3 times before they matched the emissions of disposable oximeters. Further, we estimate that if a similar-sized ED that was using disposable oximeters changed entirely to reusable ones, they would reduce their related emissions by up to 7,117.6 kgCO_2_ annually. This is nearly equivalent to the energy used by one U.S. household per year.^27^

For single-use oximeters, the production and transportation phases contributed the greatest environmental burden. However, the main source of GHG emissions for the reusable oximeters was due to its cleaning phase, which contributed 99% of its daily GHG emissions, although this still represented a fraction of the GHG emissions created by single-use oximeters. Shared equipment can become colonized with multi-drug resistant bacteria such as methicillin-resistant *Staphylococcus aureus* and vancomycin-resistant enterococci^28^ Since iatrogenic spread of communicable diseases remains a significant concern in Canadian hospitals,^29^ ^,^ ^30^ thorough cleaning of reusable devices is essential. The methods employed at this hospital are approved by the infection control service and meet the instructions for use from the manufacturer. To our knowledge, there is no practical alternative cleaning process with a lower carbon footprint that could be used.

Beyond the cleaning phase, looking at specific components of the devices themselves, the cable had the greatest environmental impact in the production phase. This may be due to a variety of factors including the large weight of the cable relative to other components of the oximeters or the high proportion of polyvinyl chloride (PVC) used in the cable’s jacketing. A 2021 analysis of the environmental footprint of various types of cable jacketing found that PVC has a higher carbon footprint compared to other materials such as high-density polyethylene.^31^

The supply chain is the biggest factor in climate-changing pollution from healthcare services. It is also the hardest to mitigate, as hospitals and healthcare centers need a wide variety of materials and equipment from multiple manufacturers to provide high-quality care, and healthcare professionals have little control over the emissions associated with these materials and equipment. This contrasts with direct on-site Scope 1 GHG emissions from a combustion of fossil fuels and direct emission of waste anesthetic gases, as well as Scope 2 emissions from purchased electricity and other energy. Healthcare organizations have near-complete control over those categories of emissions, with many opportunities for reductions. As a result, we must find any way we can to reduce the impact of our purchased goods and services, which can be achieved by a sustainable approach to device procurement.

Many LCA studies have been published in healthcare comparing reusable and disposable devices. Studies investigating operating room linens,^32^ scrubs,^33^ laryngoscopes,^9^ drug trays,^34^ central venous catheter kits,^35^ laryngeal mask airways,^36^ and vaginal specula^13^ have all shown reusable items to have superior environmental performance over single-use disposables. This is the first such study looking at equipment specifically in the ED, and it provides evidence that facilities can greatly reduce their environmental impact from a very commonly used piece of equipment. Further, one previous analysis showed that by switching entirely to reusable oximeters, the cost of providing pulse oximetry was decreased by 56%.^37^ Therefore, EDs stand to benefit economically from this change as well.

Healthcare systems, such as Kaiser Permanente in California, have committed to becoming carbon neutral or net-zero in GHG emissions.^38^ This will be accomplished in part by setting sustainability targets for their procurement division, including one that 50% of their purchased products meet environmental standards by 2025.^39^ This can be a major signal to manufacturers that maintained or increased market share can be achieved by setting and achieving sustainability targets. Over time, this will reduce the healthcare system’s environmental footprint, helping to work toward better planetary health for future generations. As mentioned, reducing Scope 3 emissions from the supply chain will be challenging without effort from manufacturers and vendors. Healthcare institutions can use their power as trusted voices focused on human health to advocate for public policies that lead to improving the environmental performance of the overall energy system, including electricity grids, which will then reduce impacts from the supply chain. In the meantime, healthcare systems can work with their group purchasing organizations to obtain environmental performance data on the products they buy, giving preference to manufacturers that have strong environmental commitments and lower emissions.

## LIMITATIONS

There are several limitations in our study that must be acknowledged. First, very few patients triaged to an unmonitored room would have their vital signs repeated four additional times during their ED visit as these are typically low-acuity, stable patients. Therefore, we likely overestimated the true impact of the cleaning phase for the 12 portable monitors in our department. Second, we were unable to obtain the material composition of the cable and LED sensor for either the reusable or disposable pulse oximeters. Therefore, we included materials used in the production of a comparable product from a different manufacturer that was willing to share these data. Since the weights of these materials were also unknown, we assigned an equal weight to each material in the cable. This may have resulted in an over- or underestimation of the contribution of the cable’s materials. However, we applied the same methodology to both devices; therefore, it was unlikely to have a significant impact on our comparative results.

In addition, although the product manufacturers were able to provide most of the information for the LCA, the exact details of some of the material specifications needed to be supplemented by other sources.^8^ ^,^ ^40^ ^–^ ^43^ These production processes are likely representative of typical industrial techniques but may not exactly correspond to the methods and efficiencies of the specific factories in which our pulse oximeters are made. In the future, a more transparent reporting process of material composition and weights would help to facilitate a more robust analysis. Finally, the scope of this project was limited to a single brand of pulse oximeter, delivered to and used in a specific setting, which may limit the generalizability of these findings. Future research comparing the life cycles of multiple brands of oximeters would help to confirm whether reusable devices are universally preferred over disposable ones when it comes to greenhouse gas emissions.

## CONCLUSION

In summary, the results of our life cycle assessment found that reusable pulse oximeters in the emergency department have a two- to five-fold lower carbon footprint than their disposable counterparts. Given that the pulse oximeter is such a ubiquitous piece of medical equipment globally, healthcare-associated carbon emissions could be significantly improved with increased use of these devices over disposable oximeters.

## Supplementary Information




