# Supplementary material for: Sustainable Purchasing Practices: A Comparison of Single-use and Reusable Pulse Oximeters in the Emergency Department
Source: West J Emerg Med. 2023 Sep 25;24(6):1034–42. doi: 10.5811/westjem.58258 (PMC10754185; doi:10.5811/westjem.58258)
Supplement: Supplementary file 1 [file wjem-24-1034-s001.docx]

**Supplemental information**

**Table 1. Impact Assessment, single reusable pulse oximeter** (N.B. 1/365^th^x34 used per day in LCA**)**

| **Impact category** | **Unit** | **Total** | **Epoxy resin,** | **Silicone product** | **Steel, chromium steel 18/8** | **Poly-carbonate** | **Lead** | **Trans** | **Corr. board box** | **Reusable Cable** | **LED sensor** | **Waste paperboard** | **Waste plastic, mixture** |
| --- | --- | --- | --- | --- | --- | --- | --- | --- | --- | --- | --- | --- | --- |
| **Ozone depletion** | kg CFC-11 eq | 2.94E-08 | 2.62E-10 | 6.13E-09 | 3.64E-10 | 2.25E-10 | 2.17E-10 | 1.18E-08 | 2.59E-09 | 7.77E-09 | 2.89E-10 | -3.79E-10 | 7.62E-11 |
| **Global warming** | kg CO2 eq | 0.35437821 | 0.00166406 | 0.00888213 | 0.00688767 | 0.0844164 | 0.0013979 | 0.04985574 | 0.02715084 | 0.14478301 | 0.00578472 | -0.001741 | 0.02529671 |
| **Smog** | kg O3 eq | 0.03015894 | 9.19E-05 | 0.00049686 | 0.00041866 | 0.00336801 | 0.00012118 | 0.00831238 | 0.00177279 | 0.01445799 | 0.00038456 | -0.000288 | 0.00102255 |
| **Acidification** | kg SO2 eq | 0.00441153 | 6.75E-06 | 3.88E-05 | 3.47E-05 | 0.00026747 | 2.75E-05 | 0.00030847 | 0.00011272 | 0.00351347 | 3.16E-05 | -1.11E-05 | 8.12E-05 |
| **Eutro** | kg N eq | 0.00359564 | 5.16E-06 | 2.23E-05 | 2.24E-05 | 2.93E-05 | 1.20E-05 | 5.99E-05 | 0.00014141 | 0.00186556 | 2.48E-05 | -1.84E-06 | 0.0014146 |
| **Carcin** | CTUh | 7.83E-08 | 1.22E-10 | 4.78E-10 | 1.93E-08 | 2.75E-09 | 8.23E-10 | 2.90E-09 | 1.88E-09 | 4.87E-08 | 4.17E-10 | -9.01E-11 | 9.80E-10 |
| **Non carcin** | CTUh | 9.13E-07 | 3.73E-10 | 1.91E-09 | 4.24E-09 | 9.68E-09 | 1.68E-08 | 1.09E-08 | 6.71E-09 | 8.23E-07 | 2.14E-09 | -2.44E-10 | 3.80E-08 |
| **Respiratory effects** | kg PM2.5 eq | 0.00062134 | 1.40E-06 | 6.28E-06 | 2.16E-05 | 4.26E-05 | 3.09E-06 | 3.55E-05 | 2.21E-05 | 0.00037881 | 8.02E-06 | -1.33E-06 | 0.00010338 |
| **Ecotoxicity** | CTUe | 64.186522 | 0.02533737 | 0.10471875 | 0.35795806 | 0.15677192 | 0.3270384 | 0.37922186 | 0.34876547 | 60.44155 | 0.12776773 | -0.0085735 | 1.9259658 |
| **Fossil fuel depletion** | MJ surplus | 0.56762223 | 0.00383717 | 0.0182482 | 0.00542091 | 0.14546454 | 0.00140615 | 0.10578015 | 0.03978051 | 0.24544905 | 0.00502746 | -0.0035218 | 0.00072992 |

**Trans= Transport, freight, lorry 16-32 metric ton; Corr.=** **Corrugated board box, Eutro= eutrophication, Carcin= carcinogenics,**

**Table 2. Impact Assessment, single disposable pulse oximeter** (N.B 150 per day used in LCA)

| **Impact category** | **Unit** | **Total** | **Copper-rich material** | **Kraft paper** | **PU adhesive** | **Textile, NWP** | **Packaging film, LDP** | **Corr. board** | **LED sensor** | **Single use Cable** | **Trans** | **Waste plastic, mixture** | **Waste paper** |
| --- | --- | --- | --- | --- | --- | --- | --- | --- | --- | --- | --- | --- | --- |
| **Ozone depletion** | **kgCFC-11eq** | 1.92E-08 | 3.99E-11 | 1.62E-11 | 5.01E-10 | 2.76E-11 | 3.63E-10 | 2.01E-09 | 2.89E-10 | 9.14E-09 | 7.03E-09 | 3.67E-11 | -2.94E-10 |
| **Global warming** | **kgCO2e** | 0.15605849 | 0.00039978 | 0.00017021 | 0.00365626 | 0.00062686 | 0.0105724 | 0.02108304 | 0.00578472 | 0.07335389 | 0.02958614 | 0.01217707 | -0.00135189 |
| **Smog** | **kgO3eq** | 0.01510092 | 6.94E-05 | 2.04E-05 | 0.00025741 | 3.40E-05 | 0.00060846 | 1.38E-03 | 0.00038456 | 0.00714871 | 0.00493286 | 0.00049222 | -2.24E-04 |
| **Acid** | **kgSO2eq** | 0.00213515 | 2.52E-05 | 1.15E-06 | 1.76E-05 | 2.51E-06 | 4.22E-05 | 8.75E-05 | 3.16E-05 | 0.00171391 | 1.83E-04 | 3.91E-05 | -8.65E-06 |
| **Eutro** | **kgNeq** | 0.00182014 | 1.17E-05 | 2.37E-06 | 1.21E-05 | 1.36E-06 | 2.62E-05 | 1.10E-04 | 2.48E-05 | 0.00091671 | 3.55E-05 | 0.00068095 | -1.43E-06 |
| **Carcin** | **CTUh** | 2.95E-08 | 3.89E-10 | 1.98E-11 | 3.46E-10 | 3.90E-11 | 6.58E-10 | 1.46E-09 | 4.17E-10 | 2.40E-08 | 1.72E-09 | 4.72E-10 | -7.00E-11 |
| **Non carcin** | **CTUh** | 4.42E-07 | 6.10E-09 | 5.02E-11 | 1.06E-09 | 1.03E-10 | 1.79E-09 | 5.21E-09 | 2.14E-09 | 4.01E-07 | 6.46E-09 | 1.83E-08 | -1.89E-10 |
| **Resp** | **kgPM2.5eq** | 0.00029559 | 2.64E-06 | 2.11E-07 | 3.43E-06 | 3.95E-07 | 7.41E-06 | 1.71E-05 | 8.02E-06 | 0.00018658 | 2.10E-05 | 4.98E-05 | -1.03E-06 |
| **Ecotoxicity** | **CTUe** | 31.527692 | 0.40149568 | 0.00235243 | 0.064799 | 0.00713506 | 0.09494173 | 0.27082167 | 0.12776773 | 29.412891 | 0.2250435 | 0.92710185 | -0.0066575 |
| **Fossil fuel depletion** | **MJ surplus** | 0.27817354 | 0.00057739 | 0.00018506 | 0.00837621 | 0.0025463 | 0.03879131 | 0.03089017 | 0.00502746 | 0.13138939 | 0.06277364 | 0.00035136 | -0.0027347 |

**PU= Polyurethane, NWP= nonwoven polypropylene, LDP= low density polyethylene, Trans= transport, freight, lorry 16-32 metric ton; Corr.=** **Corrugated board box, Acid= Acidification, Eutro= eutrophication, Carcin= carcinogenics, Resp= respiratory effects**

**Table 3. Impact Assessment, whole disinfectant wipe** (N.B. ¼ wipe used in LCA and multiplied as per low-, moderate-, and high-use cleaning scenarios)

| **Impact category** | **Unit** | **Total** | **Fibre, cotton** | **Ammonium chloride** | **Isopropanol** | **Transport, freight** | **Corrugated board box** | **Polyethylene, high density, granulate** | **Waste yarn and waste textile** | **Waste paperboard** | **Waste plastic, mixture** |
| --- | --- | --- | --- | --- | --- | --- | --- | --- | --- | --- | --- |
| **Ozone depletion** | **kg CFC-11 eq** | 3.87E-10 | 6.68E-11 | 5.83E-13 | 5.20E-11 | 5.62E-11 | 1.53E-10 | 4.83E-11 | 6.70E-12 | 2.87E-12 | 1.32E-12 |
| **Global warming** | **kg CO2 eq** | 0.010233 | 0.00115787 | 8.04E-06 | 0.00130091 | 0.00023666 | 0.00173925 | 0.00168416 | 0.00070272 | 0.0029635 | 0.0004399 |
| **Smog** | **kg O3 eq** | 0.00045478 | 0.00011261 | 4.87E-07 | 6.86E-05 | 3.95E-05 | 0.00010396 | 8.63E-05 | 5.82E-06 | 1.98E-05 | 1.78E-05 |
| **Acidification** | **kg SO2 eq** | 3.93E-05 | 1.59E-05 | 3.46E-08 | 5.37E-06 | 1.46E-06 | 7.02E-06 | 6.29E-06 | 2.33E-07 | 1.56E-06 | 1.41E-06 |
| **Eutrophication** | **kg N eq** | 0.00013518 | 4.65E-05 | 4.38E-08 | 2.68E-06 | 2.84E-07 | 9.49E-06 | 2.62E-06 | 2.08E-05 | 2.82E-05 | 2.46E-05 |
| **Carcinogenics** | **CTUh** | 6.33E-10 | 2.91E-10 | 7.15E-13 | 6.63E-11 | 1.38E-11 | 1.18E-10 | 1.10E-10 | 2.58E-12 | 1.37E-11 | 1.70E-11 |
| **Non carcinogenics** | **CTUh** | 2.57E-09 | 6.33E-10 | 2.34E-12 | 2.88E-10 | 5.17E-11 | 4.34E-10 | 2.27E-10 | 3.34E-11 | 2.44E-10 | 6.61E-10 |
| **Respiratory effects** | **kg PM2.5 eq** | 8.17E-06 | 1.26E-06 | 6.33E-09 | 6.14E-07 | 1.68E-07 | 1.42E-06 | 8.28E-07 | 2.78E-08 | 2.05E-06 | 1.80E-06 |
| **Ecotoxicity** | **CTUe** | 0.16318063 | 0.05868629 | 0.00015508 | 0.01664879 | 0.00180011 | 0.02284381 | 0.01390895 | 0.00789673 | 0.00774892 | 0.03349195 |
| **Fossil fuel depletion** | **MJ surplus** | 0.01649396 | 0.00080553 | 9.50E-06 | 0.00484791 | 0.00050212 | 0.00249334 | 0.0077324 | 6.39E-05 | 2.66E-05 | 1.27E-05 |

**Table 4. EcoInvent unit processes used**

|  | **Component** | **Unit process** |
| --- | --- | --- |
| **Single use oximeter** | Housing | Copper-rich materials {GLO}\| market for copper-rich materials \| Cut-off, U  *Polyurethane adhesive {GLO}\| market for polyurethane adhesive \| Cut-off, U  Textile, nonwoven polypropylene {GLO}\| market for textile, nonwoven polypropylene \| Cut-off, U |
|  | Cable | Polyvinylchloride, suspension polymerised {GLO}\| market for \| Cut-off, U  Polyester resin, unsaturated {RoW}\| market for polyester resin, unsaturated \| Cut-off, U  *Polyurethane, flexible foam {RoW}\| market for polyurethane, flexible foam \| Cut-off, U  Aluminum, primary, ingot, at plant/RNA  Copper-rich materials {GLO}\| market for copper-rich materials \| Cut-off, U  Brass {RoW}\| market for brass \| Cut-off, U  Acrylonitrile-butadiene-styrene copolymer {GLO}\| market for \| Cut-off, U |
|  | LED sensor | Light emitting diode {GLO}\| production \| Cut-off, U  *Epoxy resin, liquid {RoW}\| market for epoxy resin, liquid \| Cut-off, U  Aluminium oxide, metallurgical {RoW}\| market for aluminium oxide, metallurgical \| Cut-off, U |
|  | Packaging | Kraft paper {RoW}\| market for kraft paper \| Cut-off, U  Packaging film, low density polyethylene {GLO}\| market for \| Cut-off, U  Corrugated board box {RoW}\| market for corrugated board box \| Cut-off, U |
|  | Transportation | Transport, freight, lorry 16-32 metric ton, EURO3 {RoW}\| transport, freight, lorry 16-32 metric ton, EURO3 \| Cut-off, U |
|  | Waste disposal | Waste plastic, mixture {RoW}\| market for waste plastic, mixture \| Cut-off, U  Waste paperboard, unsorted {RoW}\| market for waste paperboard, unsorted \| Cut-off, U |
| **Reusable oximeter** | Housing | *Epoxy resin, liquid {RoW}\| market for epoxy resin, liquid \| Cut-off, U  *Silicone product {RER}\| market for silicone product \| Cut-off, U  Steel, chromium steel 18/8 {GLO}\| market for \| Cut-off, U  Polycarbonate {GLO}\| market for \| Cut-off, U  Lead {GLO}\| market for \| Cut-off, U |
|  | Cable | Polyester resin, unsaturated {RoW}\| market for polyester resin, unsaturated \| Cut-off, U  *Polyurethane, flexible foam {RoW}\| market for polyurethane, flexible foam \| Cut-off, U  Aluminum, primary, ingot, at plant/RNA  Copper-rich materials {GLO}\| market for copper-rich materials \| Cut-off, U  Brass {RoW}\| market for brass \| Cut-off, U  Acrylonitrile-butadiene-styrene copolymer {GLO}\| market for \| Cut-off, U |
|  | LED sensor | Light emitting diode {GLO}\| production \| Cut-off, U  *Epoxy resin, liquid {RoW}\| market for epoxy resin, liquid \| Cut-off, U  Aluminium oxide, metallurgical {RoW}\| market for aluminium oxide, metallurgical \| Cut-off, U |
|  | Packaging | Corrugated board box {RoW}\| market for corrugated board box \| Cut-off, U |
|  | Transportation | Transport, freight, lorry 16-32 metric ton, euro3 {RoW}\| market for transport, freight, lorry 16-32 metric ton, EURO3 \| Cut-off, U |
|  | Waste disposal | Waste paperboard, unsorted {RoW}\| market for waste paperboard, unsorted \| Cut-off, U  Waste plastic, mixture {RoW}\| market for waste plastic, mixture \| Cut-off, U |
| **Cleaning wipes** | Wipe | Fibre, cotton {GLO}\| market for fibre, cotton \| Cut-off, U  Ammonium chloride {GLO}\| market for \| Cut-off, U  Isopropanol {RoW}\| market for isopropanol \| Cut-off, U |
|  | Packaging | Corrugated board box {RoW}\| production \| Cut-off, U  *Polyethylene, high density, granulate {GLO}\| market for \| Cut-off, U |
|  | Transportation | Transport, freight, lorry 16-32 metric ton, euro3 {RoW}\| market for transport, freight, lorry 16-32 metric ton, EURO3 \| Cut-off, U |
|  | Waste disposal | Waste yarn and waste textile {GLO}\| market for waste yarn and waste textile \| Cut-off, U  Waste paperboard {RoW}\| market for waste paperboard \| Cut-off, U  Waste plastic, mixture {RoW}\| market for waste plastic, mixture \| Cut-off, U |

***Material details supplemented by sources external to manufacturer specifications**
